# Determination of optical markers of cyanobacterial physiology from fluorescence kinetics

**DOI:** 10.1093/plankt/fbac025

**Published:** 2022-05-25

**Authors:** Emilie Courtecuisse, Kevin Oxborough, Gavin H Tilstone, Evangelos Spyrakos, Peter D Hunter, Stefan G H Simis

**Affiliations:** EOSA, Plymouth Marine Laboratory, Prospect Place, PL1 3DH Plymouth, Devon, UK; Chelsea Technologies Ltd, 55 Central Avenue West Molesey, Surrey KT8 2QZ, UK; EOSA, Plymouth Marine Laboratory, Prospect Place, PL1 3DH Plymouth, Devon, UK; University of Stirling, Stirling FK9 4LA, Scotland, UK; University of Stirling, Stirling FK9 4LA, Scotland, UK; EOSA, Plymouth Marine Laboratory, Prospect Place, PL1 3DH Plymouth, Devon, UK

**Keywords:** fluorescence kinetics, optical markers, cyanobacteria, algae, fluorometry

## Abstract

Compared to other methods to monitor and detect cyanobacteria in phytoplankton populations, fluorometry gives rapid, robust and reproducible results and can be used *in situ*. Fluorometers capable of providing biomass estimates and physiological information are not commonly optimized to target cyanobacteria. This study provides a detailed overview of the fluorescence kinetics of algal and cyanobacterial cultures to determine optimal optical configurations to target fluorescence mechanisms that are either common to all phytoplankton or diagnostic to cyanobacteria. We confirm that fluorescence excitation channels targeting both phycocyanin and chlorophyll *a* associated to the Photosystem II are required to induce the fluorescence responses of cyanobacteria. In addition, emission channels centered at 660, 685 and 730 nm allow better differentiation of the fluorescence response between algal and cyanobacterial cultures. Blue-green actinic light does not yield a robust fluorescence response in the cyanobacterial cultures and broadband actinic light should be preferred to assess the relation between ambient light and photosynthesis. Significant variability was observed in the fluorescence response from cyanobacteria to the intensity and duration of actinic light exposure, which needs to be taken into consideration in field measurements.

## INTRODUCTION

Phytoplankton blooms increase in magnitude, duration and frequency in response to nutrient enrichment and climate change (rising temperature and hydrological changes) in marine and freshwaters ecosystems (Paerl and Huisman, 2008, 2009; O’Neil *et al.,* 2012). In freshwater systems, cyanobacterial blooms are specifically associated with elevated nutrient concentrations (Paerl *et al.,* 2011; Paerl and Paul, 2012; Wurtsbaugh *et al.,* 2019). Excessive cyanobacterial blooms can change the water taste, odor and appearance and can be directly harmful to aquatic life and human health if the dominant species produce toxins (Whitton and Potts, 2000; Codd *et al.,* 2005a, 2005b). Such blooms affect ecosystem function (through oxygen depletion, increased turbidity, decreased plant growth and fish kills), animal and human health as well as industrial, agricultural and urban activities. Early detection of cyanobacterial species and subsequent monitoring of their potential to develop into blooms is essential to manage affected ecosystems. Several conventional and emerging methods have already been recognized and reported to achieve this. These include microscopic identification and quantification, toxicity analysis (Carmichael and An, 1999; Baker *et al.,* 2002; Oehrle *et al.,* 2010), genetic and genomic methods, such as qPCR (quantitative Polymerase Chain reaction) and microarrays (Pearson and Neilan, 2008; Srivastava *et al.,* 2013), and methods based in optics such as *in vivo* fluorometry (Beutler *et al.,* 2002), flow cytometry (Becker *et al.,* 2002) and imaging flow cytometry (Campbell *et al.,* 2013). The accuracy, specificity, operational cost and transferability of these methods varies, with microscopic analysis being the most specific and also most laborious and with optical methods generally not discriminating beyond group taxonomic level but allowing higher throughput of samples.

Phytoplankton fluorescence methods target the light-harvesting system and include a broad set of approaches to provide rapid and relatively robust results that are reproducible and non-destructive and can be used *in situ* (Zamyadi *et al.,* 2016). Within the phytoplankton, the eukaryotic algae and prokaryotic cyanobacteria display specific light-harvesting and fluorescence behaviors. First, light-harvesting pigments and structures are distinct between algae and cyanobacteria. Cyanobacteria (Prochlorophytes excepted) produce phycobilipigments [allophycocyanin (APC), phycocyanin (PC) and phycoerythrin (PE)], which also exist in Cryptophyta, Glaucophyta and Rhodophyta (Falkowski and Raven, 2013) but are not commonly found in abundance alongside cyanobacteria. In Cyanobacteria, Glaucophyta and Rhodophyta, these phycobilipigments are located in light-harvesting protein-pigment complexes (LHC) called phycobilisomes (PBS), attached to the thylakoid membrane (Beutler *et al.,* 2002). In other phytoplankton groups, the LHC are embedded in the thylakoid membrane and do not include phycobilipigments. In Cryptophyta, phycobilipigments may be found which are not directly attached to the LHC. Phycobilipigments organized in a PBS induce a discernible fluorescence response to excitation in wavebands targeting phycobilipigment absorption peaks, which are typically in the “green gap” between the absorption of light by chlorophylls and carotenoids. Excitation wavelengths targeting phycobilipigments are proven optimal markers to discriminate cyanobacteria from other phytoplankton (Beutler *et al.,* 2002; Gregor and Maršálek, 2005; Gregor *et al.,* 2007; Seppälä *et al.,* 2007). While glaucophytes and rhodophytes do possess PBS and their pigment fluorescence could be confused with cyanobacteria, rhodophytes are rarely found in freshwater areas and none of these groups are likely to be abundant in environments susceptible to cyanobacterial blooms (Dodds, 2002; Dittami *et al.,* 2017; Price *et al.,* 2017). A strong phycobilipigment fluorescence signal in freshwater bodies is therefore likely associated with cyanobacteria and is a suitable proxy for early warning.

Differences in the LHC structure and corresponding functioning of the light-harvesting and quenching apparatus further define fluorescence responses between the main phytoplankton groups. The energy from a single photon absorbed by a photosynthetic pigment can be lost through one of three pathways: it can be re-emitted as fluorescence, lost through heat dissipation or used to drive photochemistry. Photochemical quenching is caused by an increase of photochemistry, while non-photochemical quenching (NPQ) regroups a range of mechanisms dissipating energy as heat, some of which are not shared between algae and cyanobacteria. NPQ mechanisms found in both algal and cyanobacterial species include “state transitions,” which allow relatively rapid adjustment of the energy delivered from LHCs to Photosystem II (PSII). The photoreceptors triggering these transitions provide group-specific optical markers. NPQ induced through the orange carotenoid protein (OCP), a water-soluble protein linked with the PBS, occurs only in cyanobacteria (Wilson *et al.,* 2008) and is photo-activated by blue-green (BG) light or bright white light, changing OCP between its inactive orange form and its active red form. Kerfeld *et al.* (2017) reported 113 genomes of cyanobacteria which contained the protein OCP-1, which is present in almost every phylogenetic subclade. If this response is nearly ubiquitous in cyanobacteria, instruments could be designed to trigger a group-specific response to detect the presence of cyanobacteria in a mixed phytoplankton assemblage. Detailed spectrofluorometric studies to determine whether this mechanism can be observed as a targeted fluorescence response have not yet been reported.

The “ideal” fluorometric solution would assess both the biomass and physiology (potential for growth) of cyanobacteria in a water body in mixed phytoplanktonic communities. Such a solution does not yet exist in concept in scientific literature nor on the market. Estimating phytoplankton biomass using *in vivo* fluorescence is based on the assumption that fluorescence is proportional to chlorophyll *a* (Chla) concentration while photosynthetically available radiation (PAR) is constant, and as long as the product of the quantum yield of *in vivo* fluorescence (*φ_f_*), the Chla-specific spectrally averaged absorption coefficient of phytoplankton weighted by the irradiance spectrum (ā^*^), and the fluorescence intracellular reabsorption factor (Q_a_^*^), may be considered constant (Babin, 2008). Fluorometers giving a diagnostic assessment of (relative) biomass of cyanobacteria are at relatively low cost, can have an easy deployment method and generally give results in short time scale (minutes). However, the product φ_f_ ā^*^  Q_a_^*^ is subject to environmental, biological and physiological variability. While ā^*^ and Q_a_^*^ cannot be estimated directly from *in vivo* fluorescence, active fluorescence methods are able to measure the quantum yield of fluorescence.

Active fluorescence, previously established using flash-stimulated techniques, measure the photochemical efficiency of the sample while saturating it with light (Huot and Babin, 2010). This is done via the determination of the minimum quantum yield of fluorescence *F_o_* (in dark-adapted state, all reaction centers opened) maximum quantum yield of fluorescence *F_m_* (all reaction centers closed) and the variable fluorescence *F_v_*, which is the difference between *F_o_* and *F_m_*. Two instrument types are widely used for the active, saturating fluorescence approach: those which saturate PSII over multiple turnovers (reaction centers open and close repeatedly), such as pulse amplitude modulation fluorometers, and those that are able to achieve the same within a single turnover of the photosystem using order-of-magnitude higher excitation intensity, such as fast repetition rate fluorometers and other well-characterized fluorometers which capture the rise of fluorescence in the microsecond scale. Single-turnover fluorometers provide the added advantage of running samples at a higher frequency, interpreting the rate of fluorescence induction as a measure of the functional absorption cross section of photochemistry as well as the kinetics of fluorescence relaxation, which can reveal the state of NPQ of the sample. Active fluorometers include predominantly bench-top instruments, but until recently, few were designed with sensitivity to cyanobacteria in mind, and the single-turnover category of instruments required relatively more costly electronics.

Bringing down the cost of fluorometric solutions while optimizing their specificity to cyanobacteria physiology remains a challenge. Several factors should be considered to optimize fluorometer design, starting from a suitable combination of excitation and emission wavebands to achieve specificity, actinic light spectral properties and the range of excitation and actinic light intensity to reliably induce and saturate photosynthetic responses as well as other engineering challenges such as temperature and flow control. To date, these design steps have been guided foremost by technically feasibility and not by what would provide the optimal separation of signals from algae and cyanobacteria.

Current fluorometers which do target the bio-optical properties of cyanobacteria have excitation wavebands targeting PE and/or APC and record Chla PSII emission. Wavebands targeting PC, which exists in all cyanobacteria, tend to be centered at shorter wavelengths (e.g. 590 nm) than the peak absorption of the pigment (615 nm) to avoid cross-talk with emission detection windows and to limit cross-excitation of Chla. While the selection of excitation bands to target cyanobacteria-specific pigments is relatively straightforward, the interpretation of the PSII response is not. It is known that, in cyanobacteria, >80% of Chla tends to be associated with Photosystem I (PSI) rather than PSII because Chla is not a major light-harvesting pigment serving PSII in this phytoplankton group (Mimuro and Fujita, 1977). Caffarri *et al.* (2014) also suggest that, compared to algae, the number of Chla molecules in the PSII core is lower in cyanobacteria. These findings suggest that estimation of cyanobacteria biomass based on PSII fluorescence is likely negatively biased. Reciprocally, we can hypothesize that the relationship between the concentration of Chla in a sample and the fluorescence observed from PSII versus PSI may hold information on the fraction of cyanobacteria out of the total phytoplankton biomass. The question remains to what extent these nuances are observable in nature and which fluorescence markers (notably fluorescence emission windows) could be exploited to quantify these properties among the various, changing fluorescence responses of diverse phytoplankton samples. It has generally been assumed that PSI fluorescence is non-variable (*F_v_* equal to 0) but Schreiber and Klughammer (2021) showed variable PSI fluorescence in a green algal species, a cyanobacterial species and in a light-green ivy leaf using a high-resolution time and high signal/noise ratio as well as high light intensity. Considering that a small percentage of Chla is linked to PSI compared to PSII in algae while the opposite is observed in cyanobacteria (Johnsen and Sakshaug, 1996, 2007), the discovery of PSI variable fluorescence opens the possibilities of using PSI fluorescence as an optical marker to discriminate cyanobacteria in a mixed phytoplankton assemblage.

In this study, we aim to identify an optimal set of optical markers to discriminate both biomass and physiological parameters of cyanobacteria using fluorescence responses. Both excitation and emission band optimization are considered. A secondary aim of this work is to inform the development of low cost *in situ* fluorometric techniques that can diagnostically target the physiology of cyanobacteria in natural samples using active fluorescence techniques and actinic light dosing. The low-cost perspective implies that markers which can be observed by dosing actinic light, even over relatively long timescales, are preferred to those which require highly sensitive or additional detectors since these components largely determine cost.

Fluorescence kinetics measurements obtained under a range of optical configurations (blue and orange excitations, emission windows over the 650–750 nm range, BG and broadband actinic light options) are recorded in this work during experiments lasting up to 1 hour in nutrient-replete laboratory strains of three cyanobacterial and three algal species. The dataset is characterized by high spectral resolution to inform band selection and is used to inform our analysis. In summary, we hypothesize that the temporal nature of the fluorescence response is more heterogeneous between that within the main phytoplankton groups (algae/cyanobacteria), which is similar to what is known spectral fluorescence responses. Further, we expect that the use of blue actinic light, while resulting in low electron transport for PSII, can induce NPQ in cyanobacteria over time such that observable fluorescence kinetics (e.g. OCP) can be assessed using a single actinic light color. However, the rate of these kinetics may well differ between algae and cyanobacteria, providing further optical markers to distinguish their response. Third, we expect that any predictable temporal aspects of fluorescence emission from PBS pigments, changing in relation to PSII and potentially also PSI fluorescence emission during exposure to actinic light, follow the mechanics known as state transitions in cyanobacteria, which are related to the movement of PBS toward and away from PSII. Variability and consistency in the emission response rates between phytoplankton groups to actinic light treatment would inform the selection of specific emission markers and light treatments for operational use. Finally, it is expected that actinic light exposure exhibits a larger response in the change of emission at 730 nm (associated with PSI) in cyanobacteria than in algae.

## METHOD

### Phytoplankton cultures

Phytoplankton cultures used in this study included three PC-rich cyanobacteria, two chlorophytes and one diatom species. *Synechocystis* sp. UTEX 2470 (UTEX Culture Collection of Algae at the University of Texas at Austin, Austin, USA) is a unicellular freshwater cyanobacterium isolated from Berkeley (USA). The N_2_-fixing pluricellular cyanobacterium *Anabaena cylindrica* CCAP 1403/2A (culture collection of algae and protozoa, SAMS limited, OBAN, Argyll, Scotland) was originally isolated from a pond in Surrey (UK). This strain grows photoheterotrophically following glycolate uptake (Renström-Kellner and Bergman, 1990). *Spirulina platensis* TBSH1-5 is a freshwater filamentous cyanobacterium isolated from a culture starter (Terra Biosphere, Paignton, UK).

The unicellular chlorophyte *Chlorella* sp. was isolated at Plymouth Marine Laboratory (UK) from a lake water sample taken in the Hanoi municipality in Vietnam. *Nannochloropsis oceanica* CCAP 849/10 is a marine eustigmatophyte isolated from an operational hatchery in western Norway. The unicellular diatom *Phaeodactylum tricornutum* UTEX 646 (UTEX Culture Collection of Algae at the University of Texas at Austin) was originally isolated from a rock pool in Segelskär (Finland).

Batch cultures were grown at relatively low light intensity (25 μmol m^−2^ s^−1^) at a 16/8 hour light/dark photoperiod using white, blue and red LED banks. Cultures were kept in a temperature-controlled room at 22°C.

All cultures were in their exponential growth phase when fluorescence kinetics measurements were achieved. *Synechocystis* sp. UTEX 2470 was grown in BG11 medium, *A. cylindrica* CCAP 1403/2A and *Chlorella* sp. in JM medium, *P. tricornutum* UTEX 646 and *N. oceanica* was grown in the f/2Q “quad” medium (higher nitrate and phosphate concentrations than f/2 medium) and *S. platensis* was grown in modified Zarrouk’s medium (Delrue *et al.,* 2017).

### Absorption measurements and dilution targets

Spectrophotometric measurements were carried out using a PerkinElmer (Waltham, MA, USA) model Lambda1050 spectrophotometer equipped with a 150-mm integrating sphere. Samples were placed in a 10-mm quartz cuvette at the center of the sphere to minimize the influence of light scattering on the absorbance measurement. Culture medium was used as blank reference, subtracted from the absorbance of each sample.

To achieve comparable cell densities between the experiments, we adapted the dilution targets established by Simis *et al.* (2012) who set absorbance targets of *D*(675) = 0.022 and *D*(437) = 0.043 in a 1-cm cuvette for nutrient-replete algae and cyanobacteria cultures, respectively. Absorbance targets provide roughly equivalent photosynthetic light absorption while minimizing the risk of multiple scattering by the cell suspension, as stated by Mitchell *et al.* (2002) and Werdell *et al.* (2018). Here, to allow for increased signal-to-noise ratio and faster repeated fluorescence measurements, these targets were doubled to *D*(675) = 0.044 (algae) and *D*(437) = 0.086 (cyanobacteria).

Absorption (*a*, units m^−1^) was derived from absorbance as follows:

$$ a\left(\lambda \right)=\ln (10)\times \frac{D\left(\lambda \right)}{0.01}, $$

where ln(10) converts from 10-based to a natural logarithm and 0.01 corresponds to the path length of the cuvette in meters. Absorption spectra were finally zeroed at 750 nm, following blank subtraction, to remove any remaining scattering or subtraction effects caused by minor variations between blank and sample, or cell positioning in the sphere, due to the low absorption signal of the diluted samples.

### Fluorescence measurements and experiment set-up

All samples were low light adapted (<17 μmol m^−2^ s^−1^) for at least 20 minutes prior to fluorescence measurements. Fluorescence was recorded in a 10-mm quartz cuvette in a Varian Cary Eclipse (Agilent, Santa Clara, CA, USA) spectrofluorometer. This instrument comprises a red-sensitive photomultiplier tube (PMT) detector to enhance sensitivity up to 900 nm. Samples were stirred and kept at 22°C throughout the measurements using a Peltier temperature control and a mini-stirrer unit. The spectrofluorometer uses a xenon flash lamp to provide repeated high-intensity excitation flashes. The short duration of flashes coupled with the relatively long interval between flashes prevent the saturation of PSII (Simis *et al.,* 2012). A dark signal between excitation flashes is recorded by the instrument and is used to offset the observed fluorescence. An actinic light source was placed directly above the cuvette and through a cover to keep out any other source of light. This actinic light source does not interfere with the excitation-emission measurement because the “ambient light” signal in the period between excitation flashes is already subtracted from the fluorescence signal.

Fluorescence responses are expressed here as a function of excitation and emission waveband, *F_x_*(*λ_ex_*, *λ_em_*), with *λ* being the center wavelength in nm and *F_x_* being one of *F_o_*, *F*′ or *F_m_* to indicate the dark-adapted, transitory and maximum fluorescence response, respectively. During fluorescence kinetics measurements, emission spectra were recorded every 15 seconds for 1 hour at *F*′(445, *λ*) and *F*′(615, *λ*) to target Chla and PC, respectively. Emission was recorded in 10-nm wide bands at 2-nm intervals between 500 and 750 nm for *F*′(445, *λ*) and between 635 and 750 nm for *F*′(615, *λ*). To accommodate weaker fluorescence from cyanobacteria under blue excitation, the PMT detector voltage was set to 600 V for cyanobacteria and to 400 V for algal cultures with 445-nm excitation. With 615-nm excitation, the PMT voltage was set to 400 V for cyanobacteria and to 600 V for algal species. Corrections to adjust for the detector gain are described in the next section.

**Fig. 1:**
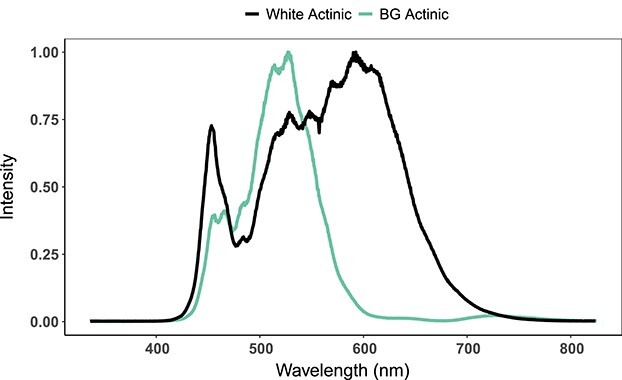
Light spectrum of the white actinic light bulb (white actinic) and light spectrum of the white actinic light bulb combined with the BG filter (BG actinic).

Actinic light was used to induce quenching, while fluorescence kinetics measurements were performed at intensities ranging from low (27–41 μmol m^−2^ s^−1^), intermediate (152–168 μmol m^−2^ s^−1^) and bright (578–597 μmol m^−2^ s^−1^) to intense (1931–1960 μmol m^−2^ s^−1^). Following initial measurements conducted over the course of 1 hour to determine the range over which temporal fluorescence responses could be discerned, further experiments were carried out under bright intensity light exposure. Light intensity was adjusted before each experiment using a Biospherical instruments Inc (San Diego, USA) PAR sensor, cross-calibrated with a calibrated spherical Micro Quantum PAR sensor, US-SQS/L (Walz). Actinic light was generated using a white LED bulb (neutral white, 540 lm, Diall) with optional BG filter (Jade 323, LEE filters) to create “white actinic” and “BG actinic” light treatments. OCP has been activated in previous studies by intense BG illumination centered at 500 nm (Tian *et al.,* 2011) and in the 400–550 nm range (Wilson *et al.,* 2008) and accordingly, in this study, the BG filter was chosen because of its high transmittance at 500 nm. White actinic produced a broad spectrum between 425 and 700 nm with peak intensity around 600 nm (Fig. 1). The BG filter combined with the white actinic light bulb induced high light intensity in the 500–545 nm range (Fig. 1). Actinic light was switched on 15 seconds after starting each experiment, i.e. after recording the first set of emission spectra on the low light-adapted sample.

### Signal normalization

Spectral calibration of the spectrofluorometer was performed according to Kopf and Heinze (1984). In summary, excitation correction factors were obtained using a quantum counter [Oxazine 1, molar absorption coefficient (641 nm) = 117 000 M^−1^ cm^−1^] in a triangular cuvette. Five excitation spectra were recorded from 200 to 700 nm (emission at 730 nm) and were averaged to obtain the excitation correction spectrum. For the emission correction, a diffusor was placed in the path from incident beam to detector, and an attenuator was placed in front of the detector. Five scans of synchronous excitation-emission measurements in the range of 200–800 nm were recorded and averaged. The excitation correction spectrum was divided by the synchronous excitation-emission spectrum to obtain the emission correction spectrum. Both correction factors were applied to all fluorescence data shown in this study.

An instrument-specific gain correction factor was obtained to normalize fluorescence data, where different PMT voltages were selected to optimize the instrument signal-to-noise levels. Fluorescence was measured using a solid standard of Rhodamine (Starna Scientific Ltd) with excitation at 445 nm and recording peak emission of the standard (≈574 nm) to obtain a correction curve as a function of PMT voltage. The correction factor (≈45.44) was applied to fluorescence obtained at a detector voltage of 400 V to align the response with fluorescence obtained at 600 V.

### Data fitting

Generalized additive models (GAM, package “mgcv” v1.8-31 for *R* v3.6.1) were used to fit localized spline regression models to the fluorescence kinetics data.

This data smoothing procedure was used to identify subtle trends in fluorescence emission to capture the dominant curvature for data visualization and to handle noise in the data, particularly in spectral regions with weak fluorescence. The performance of the GAM fit was evaluated using the adjusted *r*-squared and the proportion of the null deviance explained by the model. Relative fluorescence was calculated from fitting results (to the first value) to compare fluorescence kinetics between species. Derivatives of the GAM fitting between each time step (15 seconds) were calculated to plot the slope of fluorescence change over time.

## RESULTS

### Optical variability between phytoplankton cultures

**Fig. 2:**
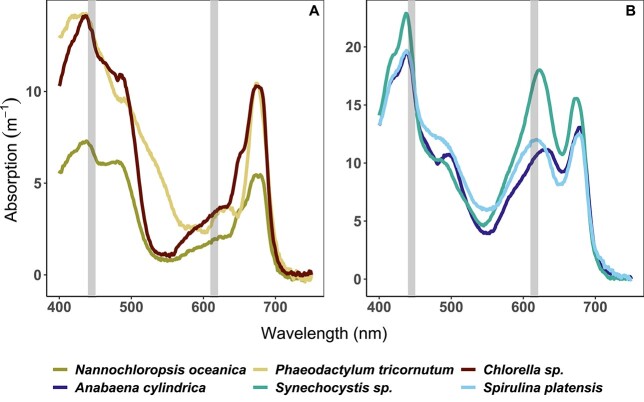
Absorption spectra of algae (**A**) and cyanobacteria (**B**) species. Gray rectangles represent two excitation wavelengths used in this paper: blue (445 nm) and orange (615 nm).

The most prominent absorption features were the distinct Chla peak absorption in the blue region at around 440 nm in all species tested and the PC absorption peak in the 610–630 nm region for the cyanobacterial species (Fig. 2). The absorption peak primarily associated with PC was found at shorter wavelengths in *S. platensis* (618 nm) and *Synechocystis* sp. (622 nm) compared to *A. cylindrica* (634 nm), suggesting variable intracellular ratios of PC, APC and Chla, which have partially overlapping absorption signatures (Fig. 2B). Absorption around the peak associated with PC exceeded that of the red Chl*a* peak at 675 nm by ~15% in *Synechocystis* sp. In the other cultures, these pigment absorption peaks were of the same order of magnitude.

In the 450–500-nm region, absorption can be primarily attributed to non-photosynthetic carotenoid pigments in cyanobacteria and the eustigmatophyte, to the presence of carotenoids and photosynthetic chlorophyll-*b* in the chlorophyte species and to carotenoids and photosynthetic chlorophyll-*c* in the diatom (Fig. 2). A significant absorption tail in the 500–560-nm region in the diatom *P. tricornutum* can be attributed to fucoxanthin (Fig. 2A). Blue-to-red absorption ratios were of similar magnitude in all cultures, confirming that culture conditions favored expression of photosynthetic over photo-protective pigments, the latter being expressed primarily in the blue part of the spectrum (Kirk, 1994).

### Low-light adapted fluorescence features of cyanobacteria and algae

Orange (*F_o_*(615, *λ*)) versus blue (*F_o_*(445, *λ*)) light excitation resulted in the typical divergent fluorescence response of algae and cyanobacteria.

In algae, fluorescence emission was consistently higher under *F_o_*(445, *λ*), while cyanobacteria exhibited several orders of magnitude higher *F_o_*(615, 685) than *F_o_*(445, 685).

**Fig. 3:**
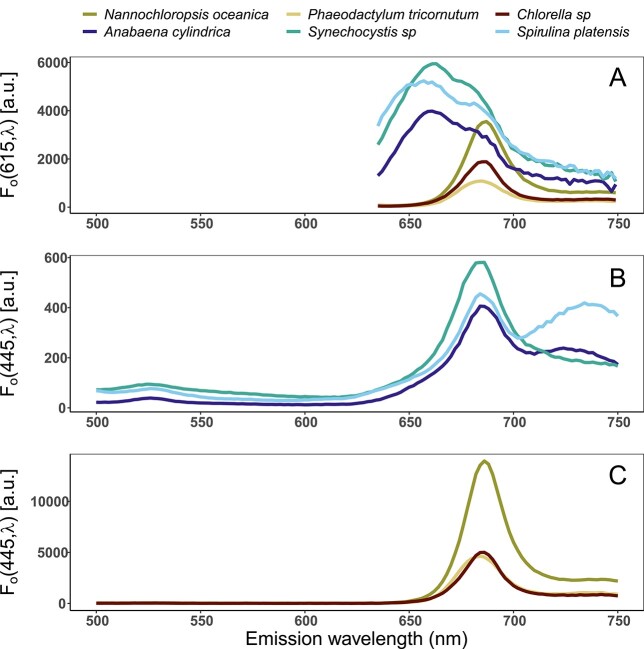
Emission spectra (*F_o_*) of all cultures under orange excitation (615 nm) (**A**), cyanobacterial (**B**) and algal (**C**) cultures under blue excitation (445 nm).

Regardless of which excitation band was used, algal cultures presented a narrow fluorescence emission band around 685 nm associated with PSII Chla and relatively low *F_o_*(*λ*, 700–750) (Fig. 3A and 3C). The near infra-red emission ranged between 16 and 23% of the PSII peak and should be mostly attributed to Chla associated with PSI Chla (see, e.g. Itoh and Sugiura, 2004). By contrast, *F_o_*(445, 685) in cyanobacteria was part of a systematically broader emission feature (Fig. 3B), which was associated with overlapping phycobilisomal fluorescence toward shorter wavelengths and a relatively higher PSI Chla contribution, ranging from 31 to 92% of the PSII peak, measured as the emission ratio of 734 over 684 nm. The *S. platensis* exhibited the highest (92%) relative fluorescence amplitude of PSI Chla compared to PSII Chl*a*. A Raman scattering feature around 525 nm (Lawaetz and Stedmon, 2009; Murphy, 2011) is discernible in the cyanobacterial emission curves (Fig. 3B), owing to a relatively low fluorescence response under blue excitation.

The *F_o_*(615, *λ*) in cyanobacteria was high in the region 640–695 nm and showed two local peaks, centered at *F_o_*(615, 660) and *F_o_*(615, 685) (Fig. 3A), corresponding to PC and APC and PSII Chla F_o_, respectively. The PC and APC fluorescence contributed, between the cyanobacterial cultures, to 30–35% of the PSII Chla peak, measured as the emission ratio [*F_o_*(615, 660) over *F_o_*(615, 684)]. Fluorescence attributed to PSI Chla was evident at wavelengths >700 nm and decreased gradually beyond 730 nm (Fig. 3A). The contribution of PSI Chla fluorescence ranged between 18 and 25% (in algae) and between 32 and 37% (in cyanobacteria) of the PSII Chla peak, (Fig. 3A) measured as the emission ratio [*F_o_*(615, 734) over *F_o_*(615, 684)].

### Actinic light exposure experiments

#### General kinetics features

**Fig. 4:**
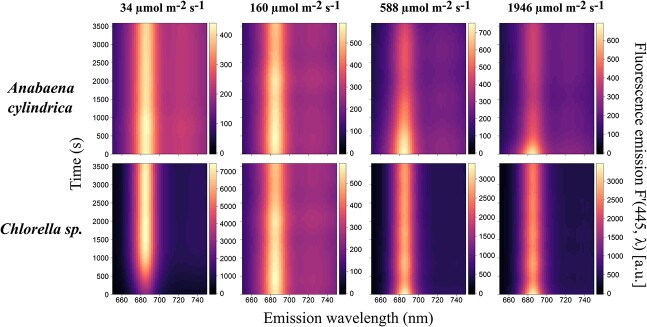
Kinetics measurements of *A. cylindrica* and *Chlorella* sp. under blue excitation (445 nm) and subjected to different intensities of white actinic light treatment. Data shown are fluorescence emission, *F*′(445, *λ*). Intensity values presented are the median of each of the following light treatment’s intensity range: low (27–41 μmol m^−2^ s^−1^), intermediate (152–168 μmol m^−2^ s^- 1^), bright (578–597 μmol m^−2^ s^−1^) and intense (1931–1960 μmol m^−2^ s^−1^).

Bright (≈588 μmol m^−2^ s^−1^) white actinic light treatment induced gradual quenching within the time frame of our experiment in *Chlorella* sp. and *A. cylindrica* (Fig. 4) and was therefore considered as suitable to assess the temporal aspects of fluorescence responses over the 1-hour duration of the experiments. By comparison, intense (≈1946 μmol m^−2^ s^−1^) white actinic light treatment induced rapid quenching at the beginning of the experiment and lower intensities induced no quenching or a rapid quenching followed by a long increase in fluorescence (Fig. 4).

**Fig. 5:**
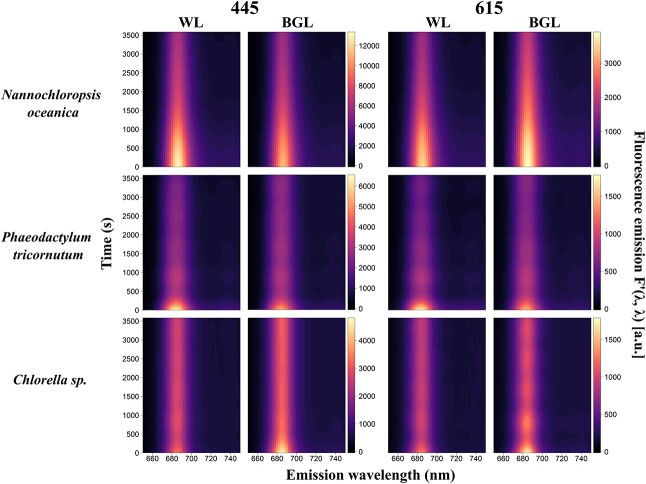
Kinetics measurements of algal species under blue (445 nm) and orange excitations (615 nm) and subjected to white actinic (WL) and BG actinic (BGL) light treatments. Data shown are fluorescence emission, *F*′(*λ*, *λ*).

**Fig. 6:**
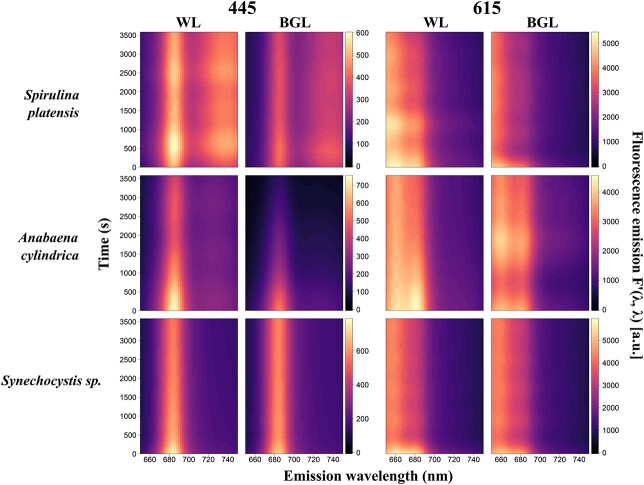
Kinetics measurements of cyanobacterial species under blue (445 nm) and orange excitations (615 nm) and subjected to white actinic (WL) and BG actinic (BGL) light treatments. Data shown are fluorescence emission, *F*′(*λ*, *λ*).

Fluorescence emission during continuous light exposure experiments remained consistently higher under *F*′(615, *λ*) compared to *F*′(445, *λ*) in cyanobacteria and for *F*′(445, *λ*) in algae (Figs 5 and 6). The specific wavebands where maximum fluorescence emission was observed differed between cyanobacterial cultures, whereas all algae showed consistent peak fluorescence emission around 685 nm (Figs 5 and 6). Under white actinic exposure, maximum fluorescence emission was of similar magnitude between the three cyanobacterial cultures, whereas *N. oceanica* had 2-fold higher fluorescence emission compared to the other two algal cultures.

#### Kinetics of algae

**Fig. 7:**
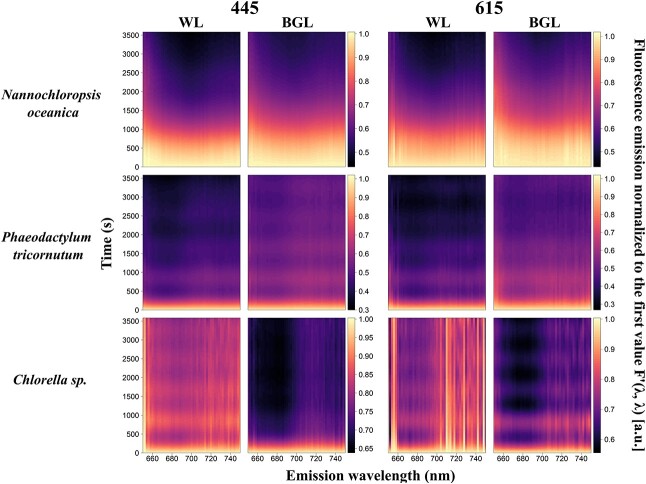
Kinetics measurements of algal species under blue (445 nm) and orange excitations (615 nm) and subjected to white actinic (WL) and BG actinic (BGL) light treatments. Data shown are fluorescence emission normalized to the first value, *F*′(*λ*, *λ*).

**Fig. 8:**
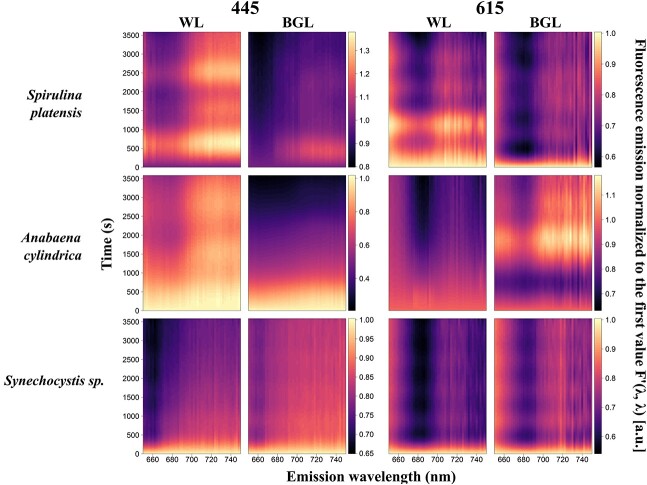
Kinetics measurements of cyanobacterial species under blue (445 nm) and orange excitations (615 nm) and subjected to white actinic (WL) and BG actinic (BGL) light treatments. Data shown are fluorescence emission normalized to the first value, *F*′(*λ*, *λ*).

**Fig. 9:**
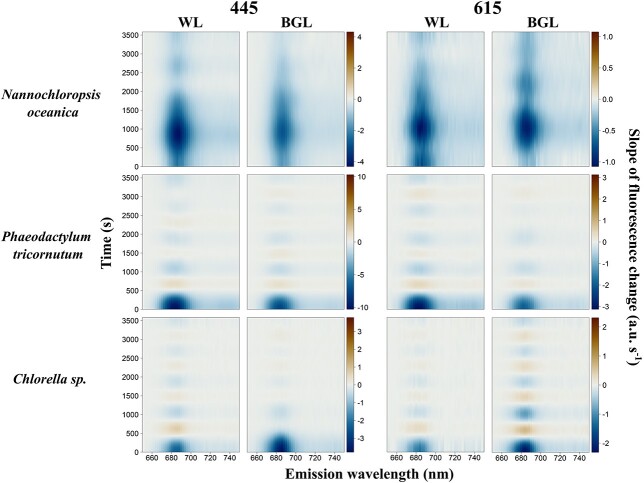
Kinetics measurements of algal species under blue (445 nm) and orange excitations (615 nm) and subjected to white actinic (WL) and BG actinic (BGL) light treatments. Data shown are the slope of fluorescence change (a.u.s^−1^).

**Fig. 10:**
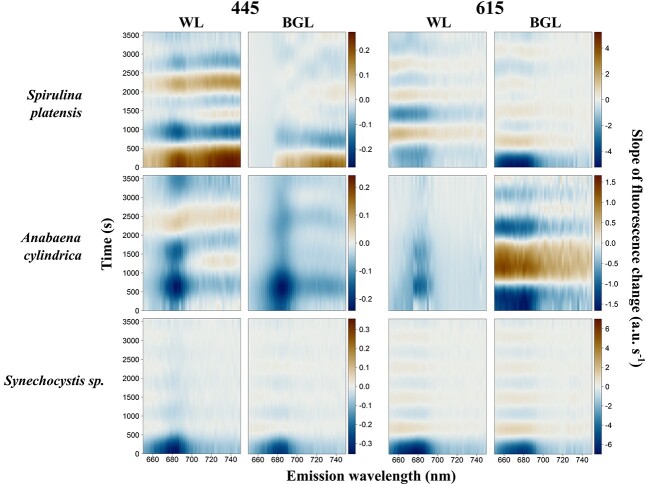
Kinetics measurements of cyanobacterial species under blue (445 nm) and orange excitations (615 nm) and subjected to white actinic (WL) and BG actinic (BGL) light treatments. Data shown are the slope of fluorescence change (a.u.s^−1^).

Algal species systematically exhibited maximum fluorescence at *F*′(*λ*, 685) which was quenched over time under both BG and white actinic exposure (Fig. 5). Quenching appeared to be spectrally uniform except in the region of the emission of PSII Chl*a* (*F*′(*λ*, 680–690)) where quenching occurred earlier. This response was most clearly observed in *N. oceanica* and *P. tricornutum* under white actinic exposure and in *Chlorella* sp. under blue actinic exposure on the relative fluorescence data (Fig. 7). The relative fluorescence for cyanobacterial cultures is shown in Fig. 8. For each emission waveband, the slope of fluorescence change is derived from data shown in Figs 5 and 6 (i.e. the derivatives of the GAM fitting between each time step) and is presented in Figs 9 and 10. These results indicate, at each emission waveband, the extent to which a photosynthetic or photoprotective mechanism was triggered, which could be considered as optical markers to differentiate cyanobacteria and algae responses to different light treatments. Fluorescence change over time (Fig. 9) was steepest (fastest) as *F*′(*λ*, 685) and steeper (faster) as *F*′(445, *λ*) than *F*′(615, *λ*). The observed changes established more quickly under white actinic light in *N. oceanica* and *P. tricornutum* but under BG Actinic exposure in *Chlorella* sp. (Fig. 9).

**Fig. 11:**
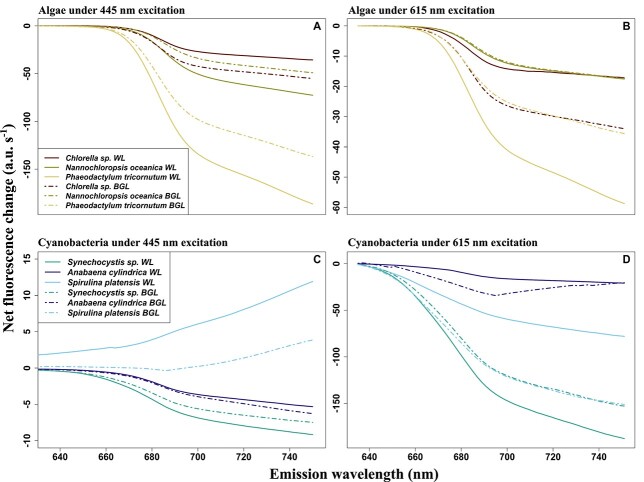
Cumulative spectral change under white actinic and BG actinic (BGL) light. The data are derived from Figs 9 and 10. (**A**) Algae under blue excitation (445 nm), (**B**) algae under orange excitation (615 nm), (**C**) cyanobacteria under blue excitation (445 nm) and (**D**) cyanobacteria under orange excitation (615 nm). Note different vertical axis scalings.

Within-group similarities in the responses to continued actinic light exposure may present at different times during the experiment as an increase or decrease in observed fluorescence. This information is contained in the slope of change-over-time spectra but is not straight forward to visualize in this context. Whether the response was net positive or negative can be shown in “cumulative spectral change” plots (Fig. 11) showing the net change per waveband over the duration of the light exposure. The steepest slopes observed between subsequent wavebands are summarized in Table I and highlight wavebands where the fluorescence emission spectrum is most likely to change during actinic light exposure. In algal species, these key wavebands were, expectedly, found in the *F*′(*λ*, 683–688) range irrespective of the light treatment. Key emission wavebands in the cyanobacterial cultures are described further below.

**Table I:** Wavebands (nm) which displayed the strongest change in fluorescence emission, determined from the cumulative spectral change (Fig. 11): the steepest slopes observed between subsequent wavebands were identified as well as the waveband where it was identified; results are shown for species exposed to white actinic and BG actinic and under blue (445 nm) and orange (615 nm) excitations

Species	White actinic		BG actinic	
	445	615	445	615
*S. platensis*	738	661	734	675
*A. cylindrica*	684	679	684	683
*Synechocystis* sp.	682	677	684	679
*N. oceanica*	688	685	686	687
*P. tricornutum*	684	685	684	683
*Chlorella* sp.	686	685	686	685

**Fig. 12:**
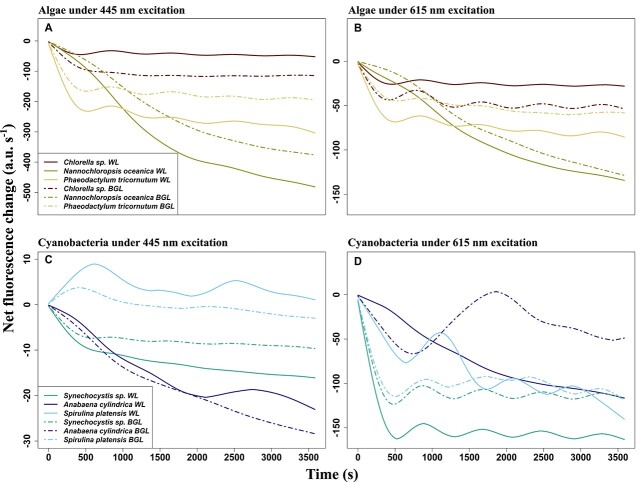
Cumulative temporal change under white actinic and BG actinic (BGL) light. The data are derived from Figs 9 and 10. (**A**) Algae under blue excitation (445 nm), (**B**) algae under orange excitation (615 nm), (**C**) cyanobacteria under blue excitation (445 nm) and (**D**) cyanobacteria under orange excitation (615 nm). Note different vertical axis scalings.

Spectral emission responses can be net zero over time when multiple photoadaptive processes occur within one emission waveband but with opposite responses. Therefore, the timing of these changes was extracted by carrying out a similar procedure as described above but along the temporal (instead of emission) axis of the fluorescence change-over-time spectra. Results of this procedure should indicate when the strongest emission changes occurred (here, considered regardless of the emission waveband) in each light treatment. The “cumulative (or net) temporal change” is shown in Fig. 12, and Table II summarizes the most prominent events of changing fluorescence emission. Cumulative temporal change (Fig. 12A and B) highlighted the differences between BG Actinic and white actinic light treatments and the two excitation wavebands. In algae, only downward trends in fluorescence emission were observed, except for short-term oscillations, notably in *Chlorella* sp. and *P. tricornutum.* These small variations are likely attributed to the GAM smoothing process, which can enhance subtle variations in the signal (GAM overfitting), although relatively slow adjustment of sample temperature or unexplained fluorescence variations during quenching cannot be ruled out.

**Table II:** Timing (s) of the strongest fluorescence emission change determined from the cumulative temporal change (Fig. 12): the steepest slopes observed between subsequent time steps were identified as well as the timing when it was identified; results are shown for species submitted to white actinic and BG actinic and under blue (445 nm) and orange excitations (615 nm)

Species	White actinic		BG actinic	
	445	615	445	615
*S. platensis*	30	1410	15	15
*A. cylindrica*	630	630	600	195
*Synechocystis* sp.	15	15	15	15
*N. oceanica*	870	1005	930	1020
*P. tricornutum*	15	15	15	15
*Chlorella* sp.	15	15	15	15

Evidence of fluorescence quenching was observed within 15 seconds after light exposure in *P. tricornutum* and *Chlorella* sp., whereas the same extent of fluorescence decrease established over 870–1020 seconds in *N. oceanica* (Figs 5, 7 and 9, Table II). Exposure to BG actinic led to slower quenching compared to white actinic in this culture (Table II). Using *F*′(615, *λ*), quenching was slower than under *F*′(445, *λ*) (Table II). This trend was not observed in the other algal species.

#### Kinetics of cyanobacteria

Relatively more diverse responses were found between the three cyanobacterial cultures. With orange excitation, the highest fluorescence emission was found around *F*′(615, 660) and *F*′(615, 685) (Fig. 6). With blue excitation, fluorescence emission was generally lower, peaking around *F*′(445, 685) and in the near infrared (*F*′(445, > 710)) in *S. platensis* (Fig. 6). The *F*′(*λ*, 650–750) under white actinic exposure yielded higher fluorescence than BG actinic exposure. This was consistent between both excitation wavebands and all cyanobacterial cultures (Fig. 6).

Quenching was predominant in *F*′(*λ*, 650–685) in *S. platensis* and *A. cylindrica* (Fig. 8). In *Synechocystis* sp., quenching prevailed at *F*′(445, 650–660) range and at *F*′(615, 685) (Fig. 8). White actinic also caused quenching at *F*′(615, 730–750) (Fig. 8).

**Fig. 13:**
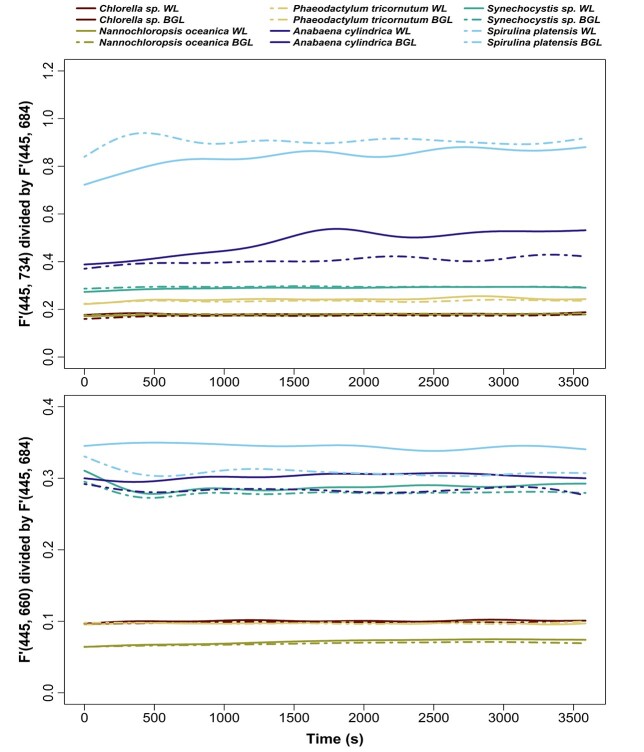
*F*′(445, 734) divided by *F*′(445, 684) (top panel) and *F*′(445, 660) divided by *F*′(445, 684) (bottom panel) over time and submitted to white actinic (WL) and BG actinic (BGL) light treatments.

In contrast to all other cultures, *S. platensis* and *A. cylindrica* exhibited both positive and negative fluorescence emission trends over the period of actinic light exposure.

Under BG actinic, *F*′(615, *λ*) of *A. cylindrica* showed quenching during the first 600 seconds of exposure, followed by an increase up to 1750 s into the experiment (Figs 6, 8 and 10). White actinic exposure of *S. platensis* similarly resulted first in a period of quenching of *F*′(615, *λ*), followed by a fluorescence increase until 1100 s after the beginning of the experiment, followed by another decrease (Figs 6, 8 and 10). The *F*′(445, *λ*) increased from the first measurement to peak only after 600 seconds. Following a period of quenching, another peak occurred around 2600 seconds (Figs 6, 8 and 10). Under BG actinic exposure, *F*′(445, *λ*) in *S. platensis* increased from the start of light exposure to reach a maximum after around 500 seconds (Fig. 8). These negative and positive emission trends were also observed on the cumulative temporal changes in *S. platensis* and *A. cylindrica* (Fig. 12C–D).

The observed fluorescence increases in *A. cylindrica* and *S. platensis* were observed in the 650–750-nm region, except for the region around *F*′(615, 685) and *F*′(445, 660–685) (Fig. 8). Short-lived variability in fluorescence was also observed in cyanobacterial cultures (Fig. 10) and could either be due to artifacts from data post-processing, slow adjustment of sample temperature or be natural fluorescence variations, as also seen in the algal cultures.

Fluorescence changes observed during actinic light exposure were more clearly expressed as *F*′(615, *λ*) compared to *F*′(445, *λ*) for cyanobacteria (Fig. 10), with the strongest fluorescence changes observed over the 661–738-nm wavelength range (Table I). These changes were observed at longer wavelengths under blue excitation compared to orange excitation (Table I). Under BG actinic, changes were found at longer wavelengths compared to white actinic exposure and differences were clearly observed under orange excitation (Table I). The *S. platensis* was an exception because changes observed under blue excitation were observed at longer wavelengths under white actinic (Table I). The changes in fluorescence were larger in *S. platensis* and *A. cylindrica* under BG actinic compared to white actinic, while the opposite behavior was observed in *Synechocystis* sp., as illustrated in the cumulative spectral changes in Fig. 11C and D.

In cyanobacteria, the strongest fluorescence changes occurred at different times and under different excitation wavebands and color of actinic light (Fig. 10, Table II). In *Synechocystis* sp., the largest fluorescence changes occurred 15 seconds into the experiment under all excitation and actinic light treatments. In the other species, the most significant fluorescence changes, under BG Actinic exposure and orange excitation, occurred earlier. For example, the most significant *F*′(615, *λ*) changes in *S. platensis* were observed at 1410 s into the experiment under white actinic compared to just 15 seconds under BG Actinic (Fig. 10, Table II).

For *A. cylindrica*, the largest fluorescence changes *F*′(615, *λ*) occurred 195 seconds after the beginning of the experiment under BG actinic compared to 630 seconds under white actinic (Table II, Fig. 10). These species presented earlier quenching under BG actinic compared to white actinic (Fig. 6).

#### Fluorescence emission ratios

The ratio of *F*′(445, 734) over *F*′(445, 684) was higher in cyanobacterial cultures than in algal cultures (Fig. 13). In all algae, the ratio was around 0.2, whereas in cyanobacteria, it varied between 0.27 and 0.93 (Fig. 13). This ratio increased over time in all cyanobacterial cultures under both white actinic and BG actinic exposure, particularly in *S. platensis* and *A. cylindrica*. The ratio of *F*′(445, 660) over *F*′(445, 684) was higher at around 0.3 in cyanobacteria compared to around 0.1 in algae (Fig. 13). Under BG actinic exposure, in cyanobacterial cultures, this ratio was lower than under white actinic exposure. In algal cultures, it increased over time of exposure as a result of ongoing quenching around 684 nm.

## DISCUSSION

### Kinetic fluorescence responses in algae and cyanobacteria

A high degree of variability in fluorescence responses during continued light exposure was found between cyanobacterial cultures, both in terms of where along the emission spectrum and when any significant transitions occurred. By contrast, algal cultures showed a high level of consistency in their fluorescence responses over time. Cyanobacterial cultures presented events of increased fluorescence along the kinetics experiments under both actinic light colors and both excitation wavelengths as well as quenching which was observed in all experiments. These results were observed in both *A. cylindrica* and *S. platensis* but were not uniform between the two species. The events occurred neither at the same time nor under the same light conditions (excitation wavelengths and actinic light color). The events of increased fluorescence in our results after quenching episodes, observed in *S. platensis* and *A. cylindrica*, suggest a recovery of NPQ mechanisms and the possibility of succession or co-occurrence of different NPQ mechanisms. To help distinguish different NPQ mechanisms, a recovery period after inducing low light exposure could be tested, but this is more suitable for an experiment employing single-turnover fluorescence instruments. Moreover, to obtain more information about photochemistry efficiency, time-resolved fluorescence measurements could be conducted.

Variability of the expression of quenching mechanisms under continued light exposure, as observed in the cyanobacterial cultures, is particularly important to consider as it highlights the difficulty to determine a generic light treatment protocol to elicit a diagnostic, targeted response in cyanobacterial fluorescence markers. On the other hand, homogeneity of algal responses is reassuring as their response can be largely predicted.

### BG actinic light and OCP-induced quenching

The choice of actinic light spectrum requires further consideration. BG actinic induced lower maximum fluorescence values than white actinic in all cyanobacterial cultures. The lower maximum emission under BG actinic is attributed to the fact that BG actinic mainly excites Chla linked to PSII, the number of Chla molecules in the PSII core complex is low (Caffarri *et al.,* 2014) and ancillary chlorophylls linked to PSII are missing or in low quantities in most nutrient-replete cyanobacteria (Mimuro, 2004). The cyanobacterial PSII excitation spectrum is weak in the blue part of the spectrum compared to longer wavebands (Yentsch and Yentsch, 1979; Schubert *et al.,* 1989; Simis *et al.,* 2012). BG actinic nevertheless appeared to induce more rapid fluorescence quenching at the beginning of the actinic light exposure experiments in two out of three cyanobacteria (*A. cylindrica* and *S. platensis*, Figs 6 and 8) under both orange and blue excitations. The BG actinic treatment was selected to highlight this feature and to explore whether it would induce NPQ quenching related to OCP protein as a photoreceptor in cyanobacteria. It has been shown that the genera *Anabaena*, *Synechocystis* and *Spirulina* all contain strains possessing genes encoding for OCP (Kerfeld *et al.,* 2017). Higher quenching observed at the beginning of the experiment in *A. cylindrica* and *S. platensis* under BG actinic compared to white actinic support the assumption of NPQ induced by OCP in these strains. Moreover, abrupt changes in the cumulative emission change shortly after the start of the BG actinic experiment (compared to white actinic) and particularly under orange excitation support the theory of OCP-induced NPQ. The quenching process was not observed in *Synechocystis* sp., and fluorescence responses were not uniform between the two cultures that did show this behavior. In algae, none of these processes were observed and there was no marked difference between BG actinic and white actinic exposure. In conclusion, using only BG actinic (as has been practiced with some active fluorometers) risks overlooking the response of cyanobacteria due to a low signal amplitude but can show an abrupt quenching reaction in part of the cyanobacteria community which has potential diagnostic use. Overall, a broad actinic light spectrum is more likely to illicit a common response in all taxa and should be part of standard measurement protocols. The ability to modulate the spectral quality of the actinic light source should be considered to further study taxonomic differences in instrument designs intended for experimental use. For example, using multiple LEDs in a bank, the blue part of the spectrum could be activated first, followed by broad white LEDs.

### PBS, PSII and PSI fluorescence emission wavelengths as emission markers

The role of the PBS pigments in cyanobacteria underlies the traditional naming of this taxonomic group as “BG algae.” Low-light acclimated and nutrient-replete cultures show a clear distinction between the photosynthetic energy harvested using PBS in the 500–650-nm range in cyanobacteria and the well-described “green gap” in photosynthetic pigmentation in algae. The most prominent fluorescence emission markers, to distinguish cyanobacteria from algae, are therefore in *F_o_*(615, *λ*) and attributed to PBS fluorescence.

Several experimental results further detail the role of PBS emission around 660 nm as well as the variability between species and groups in the PSII emission at 685 nm and PSI emission at 730 nm. Combined, these findings support potential methods to discriminate the cyanobacteria fluorescence in mixed communities, and they can be used to determine which excitation-emission pairs provide optimal targets for group-level discrimination. First, the PSI:PSII fluorescence emission ratios *F*′(445, 730)/*F*′(445, 684) and *F*′(445, 660)/*F*′(445 684) were consistently higher in cyanobacteria than algae, from low-light acclimated conditions and throughout actinic light exposure. This group-level difference may be expected to remain consistent between other cyanobacterial and algal strains and under widely ranging environmental conditions (such as light intensity and nutrient availability). This is because the assignment of pigments to PBS, PSI and PSII remains diagnostic of cyanobacteria even if the expression of individual pigments is regulated. Cyanobacteria are known to have a lower proportion of Chla linked to PSII than PSI (Mimuro and Fujita, 1977), and the majority of photosynthetic phycobilipigments in natural samples (particularly freshwater) is expected to be associated with cyanobacteria. Thus, observing relatively high PSI:PSII and PBS:PSII fluorescence emission ratios in mixed communities should be indicative of cyanobacteria presence. There is, nevertheless, reason for caution because the PSI:PSII fluorescence emission ratio in *Synechocystis* sp. was only marginally higher (0.29) than the ratio found in algae (around 0.2) in some cases. Various explanations may be found for the wide range of the PSI:PSII fluorescence emission ratio between cyanobacterial cultures. First, natural variability of the proportion of Chla attached to PSI rather than PSII could easily explain the variability between the three cultures. Differences in the efficiency of charge separations in PSII between the strains exposed to the same culturing conditions should also not be ruled out, and this could vary significantly in natural samples. Moreover, stronger quenching of fluorescence emitted by PBS under exposure to BG actinic light, as seen in Fig. 6 and 10 and Table II, in *S. platensis and A. cylindrica*, highlights the importance of PBS in discriminating cyanobacteria in mixed communities and support the choice of the emission band 660 nm.

### PSI:PSII fluorescence emission ratio under actinic light exposure

The PSI:PSII fluorescence emission ratio generally increased over time under light exposure as the result of quenching at PSII, with small variations observed in all cultures most likely attributed either to data fitting procedures or slow temperature adjustment in the sample. Larger PSI:PSII fluorescence emission ratio variations could be linked to state transitions as photosynthetic pigment was increasingly decoupled from PSII (Calzadilla and Kirilovsky, 2020). The choice of actinic light source did not differentiate cyanobacteria PSI:PSII fluorescence emission ratio responses. Further measurements of the PSI:PSII fluorescence emission ratio under different light conditions, *in situ* and in laboratory, should be carried out to further model this response. If lower PSI:PSII fluorescence emission ratios in cyanobacteria compared to algae can indeed be generalized, an emission filter centered on emission at 730 nm would be a highly useful addition to future fluorescence sensors in addition to emission bands at 660 nm to target PBS and 684 nm to target PSII.

## CONCLUSION

A range of optical configurations were tested to determine suitable optical markers to target cyanobacteria using high spectral resolution fluorescence responses. Fluorescence kinetics measured in the 650–750-nm emission range differentiated responses between cyanobacteria and algae. In particular, three emission wavelengths can be recommended to be implemented in active fluorometry of natural phytoplankton communities, centered around 660, 684 and 730 nm which, respectively, target PBS fluorescence, Chla PSII fluorescence and PSI fluorescence. The choice of blue (445 nm) and orange (615 nm) excitation channels was justified because distinct fluorescence excitation spectra are consistently observed between algae and cyanobacteria, and these wavebands provide maximum separation of absorption by the main photosynthetic pigment groups.

BG actinic treatment did not lead to common responses in the three studied cyanobacterial cultures. A broadband actinic light should be preferred, although BG light does trigger a likely OCP response which could be observable even in natural samples so that it is worth considering a modulated use of BG and white actinic light at the start of the measurement protocol. The protocol duration of 1 hour permitted, in our experiments, the observation of photoacclimation mechanisms in both algal and cyanobacterial cultures.

Nevertheless, environmental conditions (photoperiod, light intensity and nutrients’ availability) influencing phytoplanktonic species growth and light acclimation consequently induce diverse kinetics responses under a range of actinic light intensities. Those conditions have to be carefully considered before determining the duration and intensity of the actinic light to apply. The ability to change the intensity of the actinic light should, therefore, be included in fluorometer designs. Further work needs to be carried out on 734/684 and 660/684 nm fluorescence emission ratios on a wider range of freshwater species grown under different environmental conditions and excitation wavelengths, while initial results suggest taxonomically distinct responses. Extending the selection of species to additional “spectral groups,” according to the pigment composition of the peripheral antenna, is recommended. Ultimately, it may be challenging to combine multiple illumination and emission detection features in (low-cost or other) *in situ* fluorometers, but this is a promising way forward to determine cyanobacterial physiology in mixed natural communities.

## Notes

Received November 12, 2021; editorial decision April 27, 2022; accepted April 27, 2022
